# Supplementary material for: Clinical, Biochemical, and Psychological Predictors of Metabolic Syndrome in Climacteric Women
Source: Healthcare (Basel). 2025 Dec 8;13(24):3214. doi: 10.3390/healthcare13243214 (PMC12733036; doi:10.3390/healthcare13243214)
Supplement: Supplementary file 1 [file healthcare-13-03214-s001.zip › healthcare-3962128-supplementary.pdf]

**Supplementary Table S1.**

One-Way ANOVA and Tukey Post Hoc Tests for Lipid Profile Across Menopausal Stages

| Variable | PERI<br>Mean ± SD | EPM<br>Mean ± SD | LPM<br>Mean ± SD | F | p-value |
|---|---|---|---|---|---|
| CT | 181.2 ± 32.8 | 196.9 ± 31.5 | 195.4 ± 37.3 | 4.86 | 0.009 |
| C–HDL | 48.7 ± 8.5 | 55.1 ± 11.3 | 50.9 ± 11.2 | 7.12 | 0.001 |
| No–HDL | 93.4 ± 27.3 | 107.9 ± 30.6 | 113.5 ± 35.9 | 8.13 | <0.001 |

One-way ANOVA revealed significant group differences for total cholesterol, HDL, and non-HDL cholesterol ($p < 0.05$). Post hoc Tukey HSD comparisons ($\alpha = 0.05$): CT: PERI vs EPM: $\Delta = 15.65$, $p = 0.014$; PERI vs LPM: $\Delta = 14.21$, $p = 0.030$; EPM vs LPM: $\Delta = -1.44$, $p = 0.964$. C–HDL: PERI vs EPM: $\Delta = 6.31$, $p = 0.001$; PERI vs LPM: $\Delta = 2.15$, $p = 0.417$; EPM vs LPM: $\Delta = -4.16$, $p = 0.040$. No–HDL: PERI vs EPM: $\Delta = 14.48$, $p = 0.014$; PERI vs LPM: $\Delta = 20.06$, $p < 0.001$; EPM vs LPM: $\Delta = 5.57$, $p = 0.524$. CT = total cholesterol; C–HDL = high-density lipoprotein cholesterol; No–HDL = non–high-density lipoprotein cholesterol; PERI = perimenopause; EPM = early postmenopause; LPM = late postmenopause.

**Supplementary Table S2.**

S3 Results of Kruskal–Wallis and Mann–Whitney U Tests for Fasting Glucose Across Menopausal Stages

| Menopausal Stage | Median (mg/dL) | IQR Range (mg/dL) |
|---|---|---|
| Perimenopause (1) | 127.0 | 103.5–156.0 |
| Early Postmenopause (2) | 97.3 | 82.0–116.2 |
| Late Postmenopause (3) | 105.0 | 88.0–123.4 |

Kruskal–Wallis: $H(2) = 9.89$, $p = 0.007$ → Significant differences among groups ($p < 0.05$)

Pairwise Mann–Whitney U comparisons: 1 vs 2: $U = 3614.0$, $p = 0.003$; 1 vs 3: $U = 3271.5$, $p = 0.085$; 2 vs 3: $U = 2367.0$, $p = 0.094$. Overall, fasting glucose levels differed significantly across menopausal stages ($p = 0.007$). Post-hoc analysis indicated higher fasting glucose in perimenopausal women compared with early postmenopausal women ($p = 0.003$), while no differences were observed between early and late postmenopause ($p > 0.05$). IQR range: interquartile range (25th–75th percentile).

**Supplementary Table S3. Logistic Regression Models for Predictors of Metabolic Syndrome**

| Model | Variable | aOR | 95% CI | p-value |
|---|---|---|---|---|
| SM1 (Waist circumference, triglycerides, HDL-C) | Perceived stress | 0.618 | 0.433–0.882 | **0.008** |
| | Depression | 1.479 | 1.005–2.175 | **0.047** |
| | Anxiety | 0.985 | 0.664–1.463 | 0.942 |
| | | | | |
| SM2 (Blood pressure, triglycerides, fasting glucose) | Perceived stress | 1.542 | 1.072–2.219 | **0.021** |
| | Depression | 1.081 | 0.768–1.521 | 0.647 |
| | Anxiety | 1.138 | 0.784–1.653 | 0.493 |
| | | | | |
| SM3 (Waist circumference, HDL-C, fasting glucose) | Fasting glucose | 2.864 | 1.379–5.944 | **0.005** |
| | Type 2 diabetes | 2.102 | 1.082–4.083 | **0.028** |
| | Hypertension | 1.124 | 0.672–1.880 | 0.651 |
| | | | | |
| Combined model (Clinical + perceived stress) | Fasting glucose | 2.711 | 1.304–5.631 | **0.007** |
| | Perceived stress | 1.478 | 1.016–2.150 | **0.042** |

SM1 = Model including waist circumference, triglycerides, and HDL cholesterol; SM2 = Model including blood pressure, triglycerides, and fasting glucose; SM3 = Model including waist circumference, HDL cholesterol, and fasting glucose. All models were adjusted for age and relevant clinical covariates. Psychological variables were standardized (z-scores). Variance inflation factors (VIFs) were <3 in all cases, indicating no multicollinearity. Statistically significant results are shown in **bold** ($p < 0.05$).
